# Early (2 weeks) vs. late (8 weeks) initiation of highly active antiretroviral treatment (HAART) significantly enhance survival of severely immunosuppressed HIV-infected adults with newly diagnosed tuberculosis: results of the CAMELIA clinical trial

**Published:** 2011-01-10

**Authors:** François-Xavier Blanc, Thim Sok, Didier Laureillard, Laurence Borand, Claire Rekacewicz, Eric Nerrienet, Yoann Madec, Olivier Marcy, Sarin Chan, Narom Prak, Chindamony Kim, Khemarin Kim Lak, Chanroeurn Hak, Bunnet Dim, Chhun Im Sin, Sath Sun, Bertrand Guillard, Borann Sar, Sirenda Vong, Marcelo Fernandez, Lawrence Fox, Jean-François Delfraissy, Anne E Goldfeld

**Affiliations:** 1 Pneumology Unit, Internal Medicine Department, Bicêtre Hospital, Assistance Publique-Hôpitaux de Paris, Le Kremlin-Bicêtre, France; 2 Cambodian Health Committee, Phnom Penh, Cambodia; 3 European Georges Pompidou Hospital, Assistance Publique-Hôpitaux de Paris, Paris, France; 4 Institut Pasteur in Cambodia, Phnom Penh, Cambodia; 5 Agence Nationale de Recherche sur le SIDA et les hépatites virales (ANRS), Paris, France; 6 Institut Pasteur, Paris, France; 7 Khmer Soviet Friendship Hospital, Infectious Disease Department, Phnom Penh, Cambodia; 8 Donkeo Provincial Hospital, Takeo, Cambodia; 9 Médecins Sans Frontières, Phnom Penh, Cambodia; 10 Svay Rieng Provincial Hospital, Svay Rieng, Cambodia; 11 Calmette Hospital, Phnom Penh, Cambodia; 12 Siem Reap Provincial Hospital, Siem Reap, Cambodia; 13 Khmer Soviet Friendship Hospital, Pneumology Department, Phnom Penh, Cambodia; 14 Division of AIDS, NIAID, National Institute of Health, Bethesda, USA; 15 Immune Disease Institute, Harvard Medical School, Boston, USA

## Background

Tuberculosis (TB) remains the largest cause of death among people living with HIV/AIDS, especially among those with profound immunosuppression. Case-fatality among co-infected patients occurs mainly in the first months after the TB treatment initiation. Therefore, robust data regarding optimal timing of HAART initiation within this early period is critically needed.

## Methods

The CAMELIA (CAMbodian Early vs. Late Introduction of Antiretroviral drugs) clinical trial is an open-labelled randomized clinical trial designed to compare the impact upon mortality of early (2 weeks) vs. late (8 weeks) HAART initiation after TB treatment onset in treatment-naïve adults with newly diagnosed acid-fast bacilli (AFB) positive TB and CD4+ cell count ≤ 200 cells/mm^3^. Patients received standard 6-month TB treatment plus stavudine, lamivudine and efavirenz in 5 hospitals in Cambodia, 2 in Phnom Penh and 3 in province. Patients were followed for 50 weeks after the last patient’s enrollment. A log-rank test was used to compare Kaplan-Meier survival curves.

## Results

**Figure 1:**
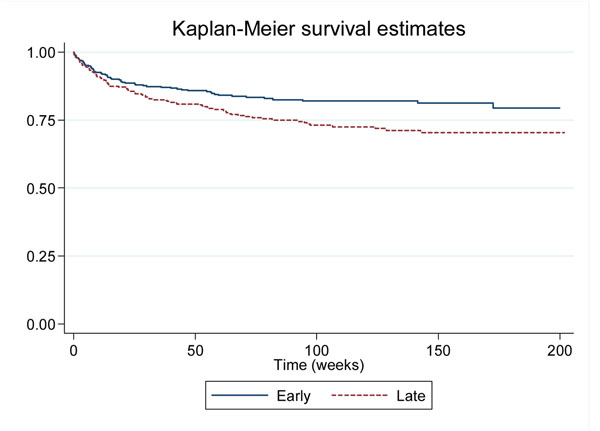


661 patients (early, n=332; late, n=329) were enrolled with a median age of 35 yrs, body mass index of 16.7 kg/m^2^, CD4+ cell count of 25 cells/mm^3^ and viral load of 5.64 log copies/ml. All AFB-positive samples including sputum in 538 (81.4%) patients, were cultured. As of May 13, 2010, 149 patients were known dead (59, early arm; 90, late arm). Enhanced survival was observed in the early arm (p=0.004, see figure). At week 50, median CD4+ gain was 114 cells/mm^3^ and was not statistically different across arms (p=0.22); 96.5% of patients had an undetectable viral load and again no difference across arms was found (0.82). Figure 1.

## Conclusion

Initiation of HAART 2 weeks after onset of TB treatment significantly improves survival in severely immunosuppressed HIV-infected adults with newly diagnosed tuberculosis.

